# Frequency and characteristics of immune-related thyroid adverse events in patients with resected stage III/IV melanoma treated with adjuvant PD-1 inhibitors: a national cohort study

**DOI:** 10.1007/s00520-024-08445-y

**Published:** 2024-04-10

**Authors:** Stine K. Christensen, Mette L. Winther, Ida J. Laursen, Freja S. Madsen, Carsten Brink, Thomas H. Brix, Eva Ellebaek, Inge Marie Svane, Frederikke S. Hansen, Charlotte Haslund, Olivia K. Laursen, Henrik Schmidt, Ida D. Larsen, Lars Bastholt, Christina H. Ruhlmann

**Affiliations:** 1 Department of Clinical Research, University of Southern Denmark, Odense, Denmark; 2 North Denmark Region, Aalborg, Denmark; 3 Region of Southern Denmark, Odense, Denmark; 4 Laboratory of Radiation Physics, Department of Oncology, Odense University Hospital, Odense, Denmark; 5 Department of Endocrinology, Odense University Hospital, Odense, Denmark; 6 Department of Oncology, National Center for Cancer Immune Therapy (CCIT-DK), Copenhagen University Hospital, Herlev, Denmark; 7 Department of Oncology, Aalborg University Hospital, Aalborg, Denmark; 8 Department of Oncology, Aarhus University Hospital, Aarhus, Denmark; 9 Department of Oncology, Odense University Hospital, Odense, Denmark

**Keywords:** Adjuvant, Immune-related adverse events, Immune checkpoint inhibitors, Immune-related thyroiditis, Melanoma, PD-1 inhibitor

## Abstract

**Purpose:**

Immune-related thyroid adverse events (irTAEs) occur frequently following immune checkpoint inhibitor (ICI) therapy. The purpose of this study is to provide knowledge about the incidence, clinical timeline characteristics, associated factors of irTAEs, and potential impact on treatment efficacy in patients with melanoma receiving adjuvant ICI therapy.

**Methods:**

A national multicenter retrospective cohort study of patients with resected stage III/IV melanoma treated with adjuvant PD-1 inhibitors between November 2018 and December 2020. Data were extracted from the Danish Metastatic Melanoma Database. The irTAEs were defined as two consecutive abnormal TSH values and subdivided into transient or persistent.

**Results:**

Of 454 patients, 99 developed an irTAE (21.8%), of these were 46 transient (46.5%) and 53 persistent (53.5%). Median time to transient and persistent irTAE was 55 and 44 days, respectively (*p* = 0.57). A hyperthyroid phase followed by hypothyroidism was seen in 73.6% of persistent irTAEs, whereas 87% of transient irTAEs developed an isolated hypo- or hyperthyroid phase. Multiple variable analysis demonstrated an association between irTAE and female sex (HR 2.45; 95% CI 1.63–3.70; *p* < 0.001), but no association with recurrence-free survival (HR 0.86; 95% CI 0.50–1.48; *p* = 0.587) or overall survival (HR 1.05; 95% CI 0.52–2.12, *p* = 0.891).

**Conclusions:**

IrTAE is a common side effect to PD-1 inhibitors primarily occurring within the first 3 months, with a high risk of persistency. Female sex is a strong predictive factor. IrTAE was not associated with improved clinical outcome.

**Supplementary Information:**

The online version contains supplementary material available at 10.1007/s00520-024-08445-y.

## Background

Immune checkpoint inhibitors (ICIs) have revolutionized cancer therapy and improved the treatment of patients with melanoma [1]. The most important immune checkpoint targets are cytotoxic T-lymphocyte antigen 4 (CTLA-4) and programmed cell death 1 (PD-1) or its ligand, programmed cell death ligand 1 (PD-L1). ICIs are designed to block the function of these, thereby promoting T-cell–mediated anti-tumor responses [2]. Nevertheless, as a result of immune activation, ICIs can lead to a distinct constellation of inflammatory side effects known as immune-related adverse events (irAEs) [3]. IrAEs can affect any organ but typically involve the skin, gastrointestinal tract, and endocrine systems [2, 4]. Most irAEs appear within the first weeks to 3 months after initiation of ICIs [5, 6], and female sex and younger age seem to be associated with higher rates of irAEs [7, 8]. Previous studies have shown that irAEs predict improved tumor response and overall survival in metastatic cancer patients treated with ICIs [1, 5, 9].

Immune-related thyroid adverse events (irTAEs) are the most common endocrine irAEs [2, 3]. Significantly higher rates of irTAEs are observed with PD-1 inhibitors relative to CTLA-4 inhibitors [10–12]. PD-1 inhibitors are successfully used in the treatment of patients with advanced melanoma, and it has been shown that the occurrence of ICI-induced irTAEs appears to be related to improved antineoplastic efficacy [13–15]. The irTAEs are often asymptomatic and consequently detected by routine laboratory tests, including thyroid stimulating hormone (TSH) and free thyroid hormones (FT3/FT4) [4]. The pathogenesis of irTAEs is widely unknown, and attempts have been made to characterize the clinical presentation [2]. The onset and pattern of irTAEs can vary; some irTAEs are characterized by a transient impact on the thyroid gland, whereas others are characterized by a persistent reduced function of the thyroid gland, requiring thyroid hormone replacement with levothyroxine [15]. In the latter, the most common clinical presentation is an initial thyrotoxic phase which subsequently, during weeks, converts into hypothyroidism [12, 16]. However, the frequency and predictors of the transient and persistent irTAEs remain unclear in an adjuvant treatment setting.

Since many patients experience irAEs and because thyroid complications occur frequently also in the adjuvant setting, it is important to provide clinicians with information to identify and understand factors that predispose to the development of irTAE as well as the impact of irTAEs on the clinical outcome.

Therefore, the aim of this retrospective national cohort study was (i) to determine the frequency and clinical timeline characteristics of transient and persistent irTAEs, (ii) to test if age and sex were associated with the risk of developing irTAEs, and (iii) to investigate associations between irTAEs with both recurrence-free survival (RFS) and overall survival (OS).

## Methods

### Study design

This is a national retrospective cohort study at the Department of Oncology of Aalborg University Hospital (AAUH), Aarhus University Hospital (AUH), Copenhagen University Hospital, Herlev (Herlev), and Odense University Hospital (OUH). Demographic information, treatment characteristics, laboratory findings, and FDG-PET/CT scan results were extracted from the Danish Metastatic Melanoma Database (DAMMED; a national database capturing oncological data on patients with melanoma in both the adjuvant and metastatic settings) [17]. Data management was completed in November 2022. The Strengthening the Reporting of Observational Studies in Epidemiology (STROBE) guideline was followed.

### Setting and treatment

Patients receiving the first treatment dose of adjuvant PD-1-inhibitor between November 28, 2018, and December 21, 2020, were included in the study. Treatment was given every fourth week in 1–13 treatment cycles. The measurement of TSH values was performed in all patients prior to the commencement of the ICI and repeated before the administration of the consecutive dose. The treatment was discontinued if the patient experienced moderate to severe (Common Terminology Criteria for Adverse Events grades 3–4) immune-related (ir)-toxicity, or due to other causes (e.g., disease recurrence) according to patient’s and/or physician’s choice. All patients were followed in the study until last seen alive or dead from any cause until March 31, 2022.

### Study participants

Patients aged ≥ 15 years with resected melanoma stage III/IV treated with an adjuvant PD-1 inhibitor were included in the study. Exclusion criteria were treatment with two different PD-1 inhibitors in the adjuvant setting, history of known thyroid illness before initiation of ICI, ir-hypophysitis, fewer than two reported TSH values, and abnormal or no available baseline TSH value. Informed consent was obtained from all participants included in the database.

### Data measurement

All laboratory values were assessed based on the respective TSH reference range (RefR) for each laboratory center; AAUH used Roche, Cobas 8000 e602, and RefR 0.3–4.5 mIU/L; AUH used Siemens ADVIA Centaur XPT and RefR 0.3–4.5 mIU/L; OUH used Roche, Cobas 8000 e602/e801, and RefR 0.3–4.0 mIU/L; Herlev used Siemens, ADVIA Centaur XP, and RefR 0.35–4.0 mIU/L, and after June 17, 2019, Herlev used Siemens Atellica IM Analyze and RefR of 0.4–4.8 mIU/L.

## Definitions

### Immune-related thyroid adverse event

Patients were categorized into two outcome groups with a further subdivision in the irTAE group inspired by previous studies [16, 18–20]: (i) no irTAE: normal TSH values or no consecutive abnormal TSH values and (ii) irTAEs: (a) transient irTAE with at least two consecutive abnormal TSH values not requiring hormone replacement or (b) persistent irTAE with at least two consecutive abnormal TSH values requiring hormone replacement, assuming that hormone replacement indicates an irreversible state of hypothyroidism. Abnormal TSH values, defined as below or above RefR depending on the respective location site, were used to detect an irTAE.

The time to develop an irTAE was defined by the time interval between the first PD-1 inhibitor dose and the first abnormal TSH value. The time to develop a hyperthyroid phase (i.e., at least one TSH value below RefR) or a hypothyroid phase (i.e., at least one TSH value above RefR) was analyzed as separate endpoints. The duration of the transient irTAE was defined as the date of the first documented abnormal TSH value until the date of the first normal TSH value without any subsequent abnormal values or until the last abnormal value. The time to start levothyroxine was defined as the date of the first abnormal TSH value until the start date of levothyroxine treatment.

### Clinical outcome

FDG-PET/CT scans were performed every 3 months during treatment. RFS was defined as the time span between the date of the first PD-1 inhibitor treatment and until recurrence of melanoma, or last scan without recurrence, determined by FDG-PET/CT scans using Response Evaluation Criteria in Solid Tumors (RECIST) [21].

OS was defined as the date of the first PD-1 inhibitor treatment until last date seen alive or date of death from any cause. A severe ir-toxicity was defined as any irAE leading to discontinuation of PD-1 inhibitor treatment. These were categorized into specific irAEs, and it was noted if the patient discontinued treatment due to one or more irAEs.

## Statistical methods

Descriptive variables were analyzed and summarized with frequency and percentage for categorical variables or as median and range/interquartile range (IQR) for continuous variables. The total incidence of irTAEs was measured during the study period, as well as the specific incidence of transient and persistent irTAEs. The time to develop an irTAE was illustrated by a Kaplan–Meier curve where patients were censored at the date of their last available TSH value. Test of difference between irTAE versus no irTAE for continuous variables was performed by Mann–Whitney *U* test, while Pearson’s chi-squared test or Fisher’s exact test was used for categorical variables. Univariate Cox proportional hazards regression analyses were performed to estimate the hazard ratio (HR) and 95% confidence interval (95% CI) for age and sex. The proportional hazard assumption was examined to check for proportionality. The significant variable from the univariate analyses was included in a multivariate analysis. A Kaplan–Meier curve with a log-rank test was used to illustrate time to an irTAE and the association with sex.

To avoid an overestimation of the RFS and OS measures in the irTAE cohort (immortal time bias), the time zero for the survival time was set equal to 6 months, thus only including patients that had survived for more than 6 months [22]. StataCorp. 2021, STATA Release 17, Statistical Software was used for all statistical analyses, and the significance level was *p* < 0.05.

A swimmer plot was created to graphically illustrate a timeline of each patient who developed an irTAE in the study.

## Results

### Baseline characteristics

A total of 554 patients were initially available for the study whereof 100 were excluded (a flow chart of patient inclusion is available in Supplementary Information, Figure SI1). Thus, 454 patients were included in the final analysis. The baseline characteristics of included patients are presented in Table 1. A total of 453 patients received nivolumab, and a single patient received pembrolizumab. Of the 454 patients, 56.6% were men, and 43.4% were women. Patients with an irTAE had a median age of 59.9 years (range 17.6–86.1) and without irTAE 62.2 years (range 21.1–86.3). A total of 63.6% of the patients with irTAE were female, whereas females only accounted for 37.7% in the group without an irTAE. No numerical differences were found in baseline characteristics between transient and persistent irTAEs.

**Table 1:** Baseline characteristics stratified by immune-related thyroid adverse event

	All patients		All irTAE	
	No irTAE	All irTAE	Transient irTAE	Persistent irTAE
	*N* = 355	*N* = 99	*N* = 46	*N* = 53
Age (years), median (range)	62.2 (21.1–86.3)	59.9 (17.6–86.1)	59.7 (17.6–86.1)	62.0 (22.2–81.0)
Sex, *n* (%)				
Male	221 (62.3)	36 (36.4)	19 (41)	17 (32)
Female	134 (37.7)	63 (63.6)	27 (59)	36 (68)
PD-1 inhibitor, *n* (%)				
Nivolumab	354 (99.7)	99 (100)	46 (100)	53 (100)
Pembrolizumab	1 (0.3)	0 (0)	0 (0)	0 (0)
TNM stage, *n* (%)				
Stage III	301 (84.8)	86 (86.9)	40 (87)	46 (87)
Stage IV	54 (15.2)	13 (13.1)	6 (13)	7 (13)
Melanoma diagnosis, *n* (%)				
Cutaneous	322 (90.7)	88 (88.9)	39 (85)	49 (92)
Unknown primary	29 (8.2)	10 (10.1)	6 (13)	4 (8)
Mucosal	4 (1.1)	1 (1)	1 (2)	0 (0)
Baseline TSH (mIU/L), median (IQR)	1.4 (1.0–1.9)	1.4 (0.9–2.3)	1.2 (0.8–2.2)	1.6 (1.1–2.6)

*irTAE* immune-related thyroid adverse event, *PD-1* programmed cell death 1, *TNM* tumor, node, metastasis, *IQR* interquartile range (25–75% percentiles), *TSH* thyroid stimulating hormone

### Frequency and characteristics of the irTAEs

A total of 99 patients (21.8%) developed an irTAE during the study period. Of these, 46 were transient irTAEs (46.5%) and 53 were persistent irTAEs (53.5%).

**Fig. 1:**
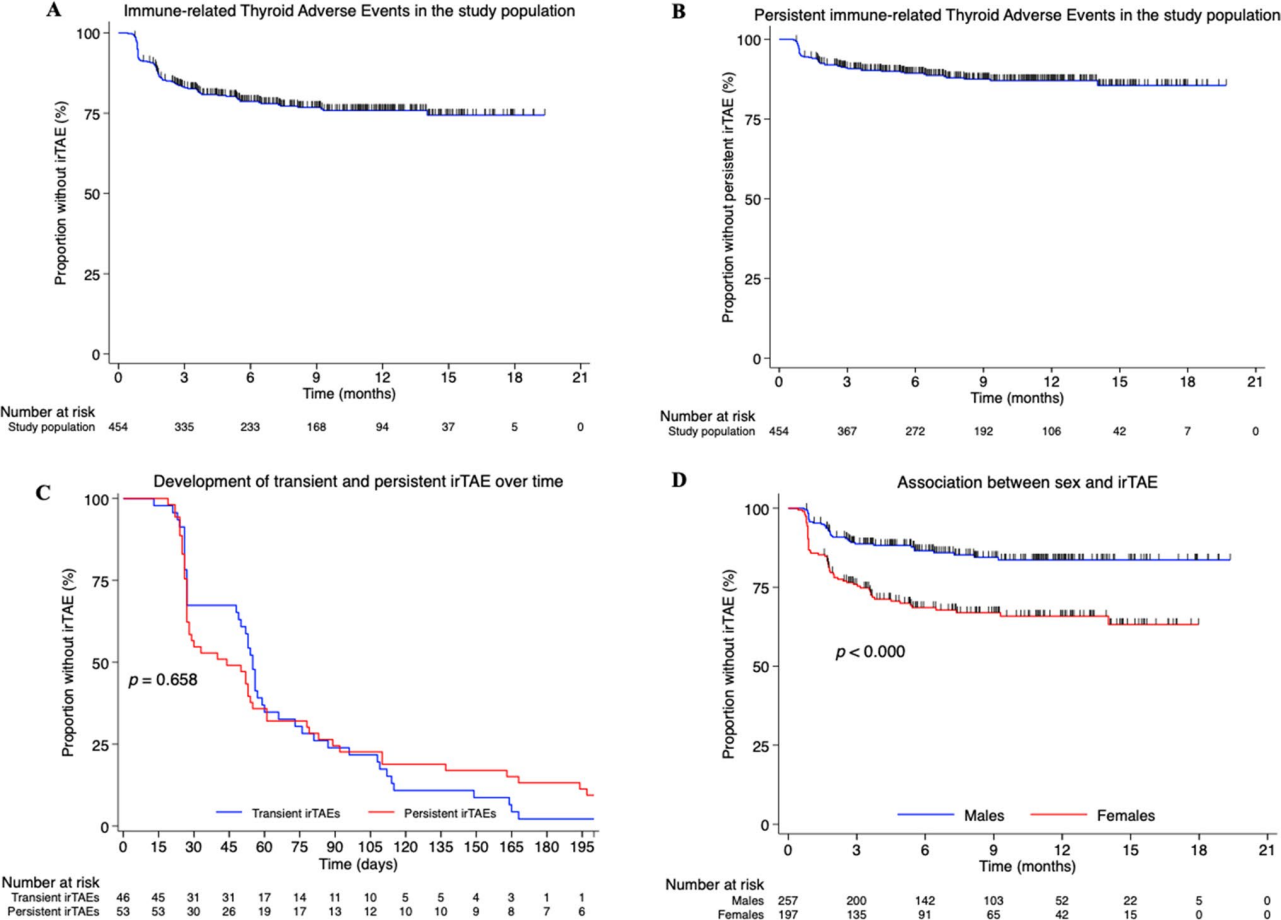
Immune-related thyroid adverse events in the study population after treatment with an adjuvant PD-1 inhibitor. **A** All irTAEs in the study population. **B** Persistent irTAEs in the study population. **C** Time to develop transient and persistent irTAEs. **D** Association between sex and irTAEs in the study population. Abbreviations: irTAE, immune-related thyroid adverse event; PD-1, programmed cell death 1

**Fig. 2:**
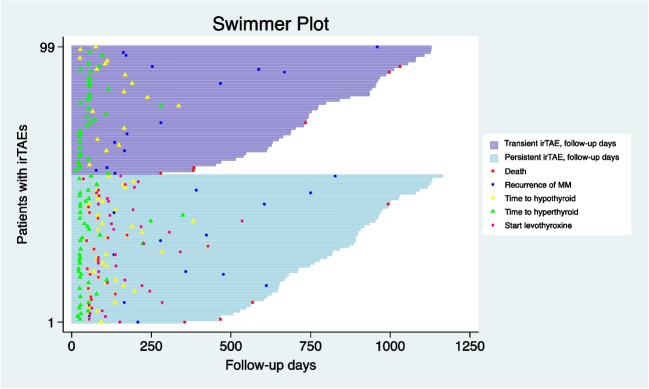
Swimmer plot of the 99 patients with an immune-related thyroid adverse event. The purple bars represent transient irTAEs, and the blue bars represent persistent irTAEs. Each bar represents an irTAE patient with symbols along each bar: time to death, time to recurrence of MM, time to first hypothyroid TSH value, time to first hyperthyroid TSH value, and time to start hormone replacement with levothyroxine after first PD-1 treatment cycle. Abbreviations: irTAE, immune-related thyroid adverse event; MM, malignant melanoma; PD-1, programmed cell death 1

For patients with an irTAE event, the median time to develop transient and persistent irTAEs was 55 days and 44, respectively (*p* = 0.57; Table 2). IrTAEs reached a plateau after 6 months (Fig. 1A, B), and no significant difference over time was found between transient and persistent (Fig. 1C). There was no statistical significant difference between transient and persistent irTAEs in time to develop hyper- and hypothyroid phases. Of persistent irTAEs, 39 (73.6%) developed a hyperthyroid phase followed by a hypothyroid phase, whereas 40 (87%) transient irTAEs had either an isolated hyper- or hypothyroid phase. Persistent irTAEs had a median of 57 days to start hormone replacement. The duration of the transient irTAE had a median of 84 days (Table 2). The swimmer plot illustrates the clinical differences of each patient with an irTAE divided into transient and persistent (Fig. 2).

**Table 2:** Comparison of clinical timeline characteristics between transient and persistent immune-related thyroid adverse event

	All irTAE		*p*-value
	Transient irTAE *N* = 46	Persistent irTAE *N* = 53	
Time to irTAE (days), median (range)	55 (13–280)	44 (19–427)	0.57
Levothyroxine treatment, *n* (%)	0 (0%)	53 (100%)	
Time to start levothyroxine after first abnormal TSH value (days), median (IQR)		57 (29–63)	
Duration of irTAE* (days), median (IQR)	84 (57–140)		
Time to hyperthyroid (days), median (range)	53 (13–280)	28 (19–349)	0.16
Time to hypothyroid (days), median (range)	109 (26–336)	84 (36–447)	0.48
The phases of the irTAE, *n* (%)			
Hyperthyroid + hypothyroid	6 (13)	39 (73.6)	** < 0.001**
Hypothyroid + hyperthyroid	0 (0)	1 (1.9)	
Isolated hyperthyroid	29 (63)	2 (3.8)	** < 0.001**
Isolated hypothyroid	11 (24)	11 (20.7)	0.81

Statistically significant *p*-values are highlighted in bold

*irTAE* immune-related thyroid adverse event, *IQR* interquartile range (25–75% percentiles), *TSH* thyroid stimulating hormone

^*^10 patients with transient irTAE remained with abnormal TSH values at the end of the study period

The follow-up time in days until last seen alive was statistically significantly shorter for patients without an irTAE compared to those with an irTAE (*p* = 0.014); however, the numerical difference was small (Table 3). Furthermore, there was a statistically significant difference in days until the last available TSH value with a median of 317 days in patients with an irTAE and a median of 250 days in patients without an irTAE (*p* < 0.001).

**Table 3:** Clinical differences between patients with and without immune-related thyroid adverse event and further between transient and persistent immune-related thyroid adverse event

	All patients		*p*-value	All irTAE		*p*-value
	No irTAE	All irTAE		Transient irTAE	Persistent irTAE	
	*N* = 355	*N* = 99		*N* = 46	*N* = 53	
Number of treatment cycles, median (range)	10 (1–13)	12 (1–13)	0.650	9 (1–13)	12 (2–13)	0.057
Time to recurrence of MM, days from 1 cycle, median (IQR)	244 (130–394)	253 (150–476)	0.346	171 (136–467)	375 (165–605)	0.326
Follow-up days, median (range)						
Until last seen alive or death	730 (60–1214)	802 (280–1165)	**0.014**	763 (280–1130)	841 (355–1165)	0.632
Follow-up days, median (range)						
Until last laboratory value	250 (23–588)	317 (56–598)	** < 0.001**	296 (56–598)	328 (75–532)	0.311

*irTAE* immune-related thyroid adverse event, *irAE* immune-related adverse event, *IQR* interquartile range (25–75% percentiles), *MM* malignant melanoma

Few differences were seen between the outcome groups with respect to dose delay of the PD-1 inhibitor, or any toxicities during the treatment period (Table 4). The same proportion of patients with and without an irTAE, approximately 30%, discontinued treatment due to toxicity. The outcome groups showed no difference in relation to specific toxicities.

**Table 4:** Differences in treatment and ir-toxicities between patients with and without immune-related thyroid adverse events and further between transient and persistent immune-related thyroid adverse events

	All patients		All irTAE	
	No irTAE	All irTAE	Transient irTAE	Persistent irTAE
	*N* = 355	*N* = 99	*N* = 46	*N* = 53
≥ 1 reduction or delay in treatment, *n* (%)				
No	264 (74.4)	66 (66.7)	29 (63)	37 (70)
Yes	91 (25.6)	33 (33.3)	17 (37)	16 (30)
Reason for reduction or delay in treatment^a^, *n* (%)				
Doctor wish	23 (6.5)	5 (5.1)	2 (4.3)	3 (5.7)
Patient wish	0 (0)	1 (1.0)	0 (0)	1 (1.9)
Toxicity	37 (10.4)	14 (14.1)	7 (15.2)	7 (13.2)
Infusion-related toxicity	7 (2.0)	2 (2.0)	1 (2.2)	1 (1.9)
Other cause	37 (10.4)	15 (15.2)	8 (17.4)	7 (13.2)
Reason stopping treatment, n (%)				
Treatment finalized	153 (43.1)	52 (52.5)	21 (46)	31 (58)
Death	1 (0.3)	0 (0.0)	0 (0)	0 (0)
Recurrence of MM	74 (20.8)	15 (15.2)	8 (17)	7 (13)
Severe overall ir-toxicity	110 (31.0)	30 (30.3)	17 (37)	13 (25)
Other cause	17 (4.8)	2 (2.0)	0 (0)	2 (4)
Reason stopping treatment if due to specific toxicities^b^, *n* (%)				
Thyroiditis	0 (0)	7 (7.1)	2 (4.3)	5 (9.4)
Hepatitis	15 (4.2)	5 (5.1)	5 (10.9)	0 (0)
Colitis	16 (4.5)	3 (3.0)	1 (2.2)	2 (3.8)
Meningitis	1 (0.3)	0 (0)	0 (0)	0 (0)
Myocarditis	1 (0.3)	3 (3.0)	2 (4.3)	1 (1.9)
Pneumonitis	7 (2.0)	0 (0)	0 (0)	0 (0)
Nephritis	7 (2.0)	0 (0)	0 (0)	0 (0)
Arthritis	8 (2.3)	1 (1.0)	1 (2.2)	0 (0)
Pancreatitis	0 (0)	1 (1.0)	1 (2.2)	0 (0)
Skin toxicity	10 (2.8)	2 (2.0)	1 (2.2)	1 (1.9)
Neuropathy	6 (1.7)	1 (1.0)	1 (2.2)	0 (0)
Myositis	3 (0.8)	3 (3.0)	2 (4.3)	1 (1.9)
Infusion-related toxicity	0 (0)	1 (1.0)	1 (2.2)	0 (0)
Stopping treatment due to > 1 specific toxicities, *n* (%)	8 (2.3)	6 (6.1)	2 (4.3)	4 (7.5)

*irTAE* immune-related thyroid adverse event, *MM* malignant melanoma

^a^Patients may have several causes for treatment delay

^b^Patients may have several toxicities

### Predictors for developing an irTAE

The female sex was statistically significantly associated with a greater risk of developing an irTAE (HR 2.52; 95% CI 1.67–3.80; *p* < 0.001) (Fig. 1D). Age was statistically significantly associated with a lower risk of developing irTAEs (HR per 10 years increase 0.86; 95% CI 0.75–0.98; *p* = 0.024). Performing a multivariate Cox proportional hazard regression with sex and age, sex proved to be an independent variable (HR 2.45; 95% CI 1.63–3.70; *p* < 0.001), whereas age was borderline significant (HR 0.88; 95% CI 0.77–1.001; *p* = 0.050).

### Clinical outcome associations

**Fig. 3:**
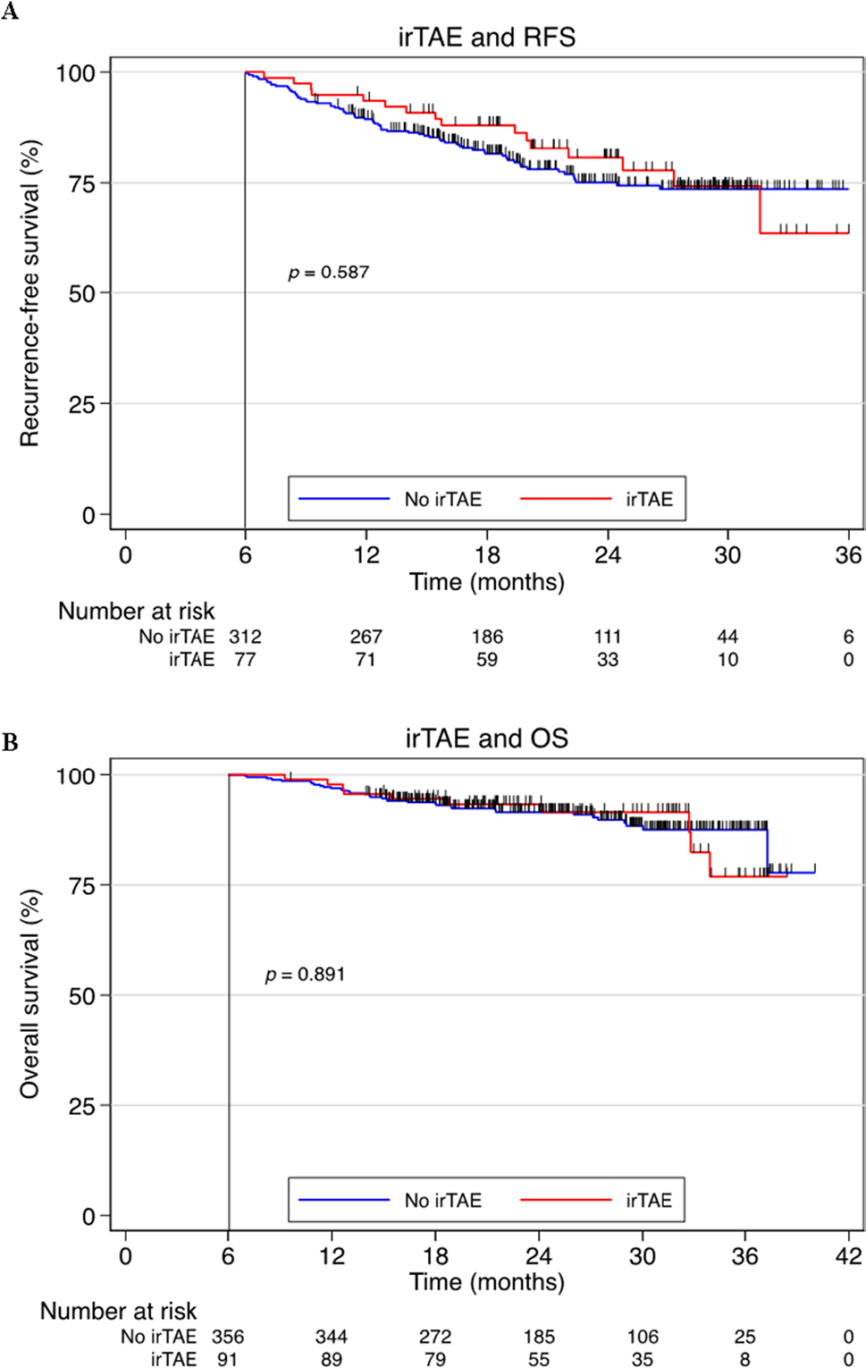
Associations between immune-related thyroid adverse events in the study population with both recurrence-free survival and overall survival after treatment with an adjuvant PD-1 inhibitor. **A** Recurrence-free survival among patients with and without immune-related thyroid adverse events. **B** Overall survival among patients with or without immune-related thyroid adverse events. The survival curves are set to start after 6-month follow-up to avoid immortality bias. Abbreviations: *irTAE* immune-related thyroid adverse event, *RFS* recurrence-free survival, *OS* overall survival

There was no statistically significant association between irTAE and RFS (HR 0.86; 95% CI 0.50–1.48; *p* = 0.587) and no statistically significant association with OS (HR 1.05; 95% CI 0.52–2.12, *p* = 0.891) (Fig. 3A, B). A numerical difference in time to recurrence of melanoma between transient and persistent was found with a median of 171 and 375 days, respectively.

### Missing TSH values

There were no statistically significant differences in baseline characteristics and number of treatment cycles when comparing patients excluded due to missing TSH values with patients included in the study. There was a statistically significant difference in the follow-up number of days until last seen alive, inherent to the fact that the majority of patients excluded due to missing TSH values started adjuvant treatment close to the cutoff date of the study (Supplementary Information, Table SI1-2).

## Discussion

To our knowledge, this is the first study to report on the clinical timeline and associated factors for developing irTAEs in patients with resected stage III/IV melanoma treated with adjuvant PD-1 inhibitors.

Previous research has demonstrated a wide range in the incidence of irTAEs among cancer patients treated with ICIs, probably given the variable assessment methods in detecting the irTAEs [12–15, 20]. We found that the development of irTAEs in patients with stage III/IV melanoma treated with adjuvant PD-1 inhibitors was common during the first 3 months, which is comparable to other studies in a metastatic setting [13, 15, 20]. Our findings are in line with the current understanding of the development of irTAE, starting with a hyperthyroid phase, which either recovers or progresses to persistent hypothyroidism [2, 12]. The current study detected 22 patients with an irTAE with a hypothyroid phase without a prior hyperthyroid phase. This could be explained by the fact that TSH values were measured every 4 weeks, and a potential hyperthyroid phase could have been missed, but factors such as degree/burden of thyroid inflammation and presence of underlying thyroid autoimmunity may also play a role. Among the transient irTAEs, 29 patients had no detected hypothyroid phase, which is similar to what others have reported [15, 20]. Overall, this suggests different degrees of severity of the irTAEs according to reversibility [15, 20]. However, mechanisms involved in developing irTAEs have not been fully elucidated [14].

The present study identified female sex and younger age as predictors of developing an irTAE, which is in accordance with the result of Muir et al., although both monotherapy and combination therapy with ICIs were included in their study [15]. The higher risk of irTAEs for females is likely to be due to a preponderance of autoimmune diseases in females compared to males [15] and the borderline significance of younger age as an independent risk factor for irTAE in unknown, but it has been suggested that it could at least partly be explained by relationship between thyroid diseases and younger women [15].

Two large systematic reviews and meta-analyses concluded that the occurrence of ICI-induced irAEs overall was significantly associated with longer progression-free survival (PFS) and OS in the metastatic setting [1, 5]. However, Sun et al. clarify in the summary analysis that specifically irTAEs were not associated with a favorable PFS and OS outcome [1]. This is consistent with the results of the current study and other research [23]. Eggermont et al. found that occurrence of an irAE was associated with a longer RFS in patients receiving pembrolizumab in the adjuvant setting (HR, 0.61; 95% CI, 0.39–0.95; *p* = 0.03) in both men and women, and comparable results were found when only endocrine irAEs were considered [24]. Additionally, other studies have reported better OS among patients with melanoma who developed an irTAE [13–15]. Furthermore, Muir et al. showed that overt thyrotoxicosis was associated with longer PFS [15]. In the current study, the time to recurrence of melanoma, albeit not statistically significant, was shorter in transient compared to persistent irTAEs. More studies are needed to investigate whether persistent irTAEs could be a surrogate marker of a more robust immune response to ICIs. Furthermore, patients with irTAEs were not more likely to discontinue treatment due to toxicity, thus indicating that irTAEs are not correlated with a higher toxicity profile.

A strength of this multicenter study was the use of a national sample size with blood samples collected prospectively. This allowed us to characterize a clinical timeline of the irTAE. The detection of the irTAE was based on abnormal TSH values since the development of irTAE in many cases does not result in clinical symptoms or signs of thyroid dysfunction [2, 4, 10].

Measurement of TSH values was analyzed with different assays. Despite this, the study managed to investigate the specific TSH RefR at the different hospitals during the given study period. This increases the precision in the detection of abnormal TSH values and, therefore, the accuracy of detected irTAEs.

To prevent misclassification and a possible overestimation in the number of detected irTAEs in the cohort, patients with known thyroid risk factors and ir-hypophysitis were excluded. Furthermore, patients excluded due to missing TSH values were not demographically or clinically different from patients included, which reduces the risk of selection bias.

Importantly, the present study controlled for time-dependent variables by taking immortality bias into account.

The current study has limitations inherent to the retrospective design. Follow-up data was inconsistent between the outcome groups. Patients with irTAEs were followed with blood samples more frequently, most likely due to the need for close monitoring of patients with irTAEs.

Relevant risk factors such as thyroid peroxidase antibodies (TPOAb), TSH receptor antibodies (TRAb), thyroglobulin antibodies (TgAb), and pre-existing autoimmune diseases were not collected. Studies have reported a positive association between TPOAb and/or TgAb and the incidence of irTAEs [15, 18]. Other studies have hypothesized that patients with pre-existing autoimmune diseases have a different immune response and consequently an enhanced risk of developing irAEs [6, 7, 25]. Overall, this limits the external validity of the study. Furthermore, information on the corresponding serum levels of the thyroid hormones FT3/FT4 could have provided a greater insight into the clinical impact of the irTAE.

## Conclusion

This multicenter cohort study is the first to provide a unique description of the clinical timeline and associated factors for the development of irTAE induced by adjuvant PD-1 inhibition in patients with melanoma. The irTAE is a common side effect most frequently occurring during the first 3 months of treatment, and the risk is higher for female and younger patients. Furthermore, this study suggests different degrees of severity according to reversibility with the division in transient and persistent irTAEs. The study did not find an association between irTAE and improved RFS or OS. Future prospective studies are needed to understand the pathogenesis, additional predictors, and potential preventive measures for the development of irTAEs and further investigate competing risk factors in relation to clinical outcome in the adjuvant setting of patients with melanoma.

### Supplementary Information

Below is the link to the electronic supplementary material.

Supplementary file1 (DOCX 275 KB)

## Data Availability

No datasets were generated or analysed during the current study.

## References

[1] Sun Q, Sun H, Wu N (2022). Patients with melanoma treated with immune checkpoint inhibitors who had non-thyroid endocrine and skin immune-related adverse events have better prognosis: a systematic review and meta-analysis. Front Oncol.

[2] Iyer PC, Cabanillas ME, Waguespack SG (2018). Immune-related thyroiditis with immune checkpoint inhibitors. Thyroid.

[3] Kennedy LB, Salama AKS (2019). A review of immune-mediated adverse events in melanoma. Oncol Ther.

[4] Sznol M, Postow MA, Davies MJ (2017). Endocrine-related adverse events associated with immune checkpoint blockade and expert insights on their management. Cancer Treat Rev.

[5] Fan Y, Xie W, Huang H (2021). Association of immune related adverse events with efficacy of immune checkpoint inhibitors and overall survival in cancers: a systemic review and meta-analysis. Front Oncol.

[6] Ramos-Casals M, Brahmer JR, Callahan MK (2020). Immune-related adverse events of checkpoint inhibitors. Nat Rev Dis Primers.

[7] Liu X, Shi Y, Zhang D (2021). Risk factors for immune-related adverse events: what have we learned and what lies ahead?. Biomark Res.

[8] Wong SK, Nebhan CA, Johnson DB (2021). Impact of patient age on clinical efficacy and toxicity of checkpoint inhibitor therapy. Front Immunol.

[9] Socinski MA, Jotte RM, Cappuzzo F (2023). Association of immune-related adverse events with efficacy of atezolizumab in patients with non-small cell lung cancer: pooled analyses of the phase 3 IMpower130, IMpower132, and IMpower150 randomized clinical trials. JAMA Oncol.

[10] de Filette J, Andreescu CE, Cools F, Bravenboer B, Velkeniers B (2019). A systematic review and meta-analysis of endocrine-related adverse events associated with immune checkpoint inhibitors. Horm Metab Res.

[11] Khoja L, Day D, Wei-Wu Chen T, Siu LL, Hansen AR (2017). Tumour- and class-specific patterns of immune-related adverse events of immune checkpoint inhibitors: a systematic review. Ann Oncol.

[12] Muir CA, Menzies AM, Clifton-Bligh R, Tsang VHM (2020). Thyroid toxicity following immune checkpoint inhibitor treatment in advanced cancer. Thyroid.

[13] Dawidowska A, Jagodzinska-Mucha P, Koseła-Paterczyk H et al (2022) Immune-related thyroid adverse events predict response to PD-1 blockade in patients with melanoma Cancers (Basel) 14. 10.3390/cancers14051248 10.3390/cancers14051248 PMC8909092 35267557

[14] Lima Ferreira J, Costa C, Marques B (2021). Improved survival in patients with thyroid function test abnormalities secondary to immune-checkpoint inhibitors. Cancer Immunol Immunother.

[15] Muir CA, Clifton-Bligh RJ, Long GV (2021). Thyroid immune-related adverse events following immune checkpoint inhibitor treatment. J Clin Endocrinol Metab.

[16] von Itzstein MS, Gonugunta AS, Wang Y (2022). Divergent prognostic effects of pre-existing and treatment-emergent thyroid dysfunction in patients treated with immune checkpoint inhibitors. Cancer Immunol Immunother.

[17] Ellebaek E, Svane IM, Schmidt H (2021). The Danish metastatic melanoma database (DAMMED): a nation-wide platform for quality assurance and research in real-world data on medical therapy in Danish melanoma patients. Cancer Epidemiol.

[18] Kotwal A, Kottschade L, Ryder M (2020). PD-L1 inhibitor-induced thyroiditis is associated with better overall survival in cancer patients. Thyroid.

[19] Zhang X, Wu Y, Lv J (2019). Nivolumab-induced thyroid dysfunctions in patients with previously treated non-small cell lung cancer. Interdiscip Sci.

[20] Al Mushref M, Guido PA, Collichio FA, Moore DT, Clemmons DR (2020). Thyroid dysfunction, recovery, and prognosis in melanoma patients treated with immune checkpoint inhibitors: a retrospective review. Endocr Pract.

[21] Schwartz LH, Litière S, de Vries E (2016). RECIST 1.1-update and clarification: from the RECIST committee. Eur J Cancer.

[22] Lévesque LE, Hanley JA, Kezouh A, Suissa S (2010). Problem of immortal time bias in cohort studies: example using statins for preventing progression of diabetes. Bmj.

[23] Yamauchi I, Yasoda A, Matsumoto S (2019). Incidence, features, and prognosis of immune-related adverse events involving the thyroid gland induced by nivolumab. PLoS One.

[24] Eggermont AMM, Kicinski M, Blank CU (2020). Association between immune-related adverse events and recurrence-free survival among patients with stage III melanoma randomized to receive pembrolizumab or placebo: a secondary analysis of a randomized clinical trial. JAMA Oncol.

[25] Mangan BL, McAlister RK, Balko JM (2020). Evolving insights into the mechanisms of toxicity associated with immune checkpoint inhibitor therapy. Br J Clin Pharmacol.

