# Supplementary material for: Frequency and characteristics of immune-related thyroid adverse events in patients with resected stage III/IV melanoma treated with adjuvant PD-1 inhibitors: a national cohort study
Source: Support Care Cancer. 2024 Apr 10;32(5):281. doi: 10.1007/s00520-024-08445-y (PMC11006760; doi:10.1007/s00520-024-08445-y)
Supplement: Supplementary file 1 — Supplementary file1 (DOCX 275 KB) [file 520_2024_8445_MOESM1_ESM.docx]

Supplementary Information for

*Frequency and characteristics of immune-related thyroid adverse events in patients with resected stage III/IV melanoma treated with adjuvant PD-1 inhibitors. A national cohort study*

Figure SI1

Flowchart of inclusion and exclusion criteria in the study cohort


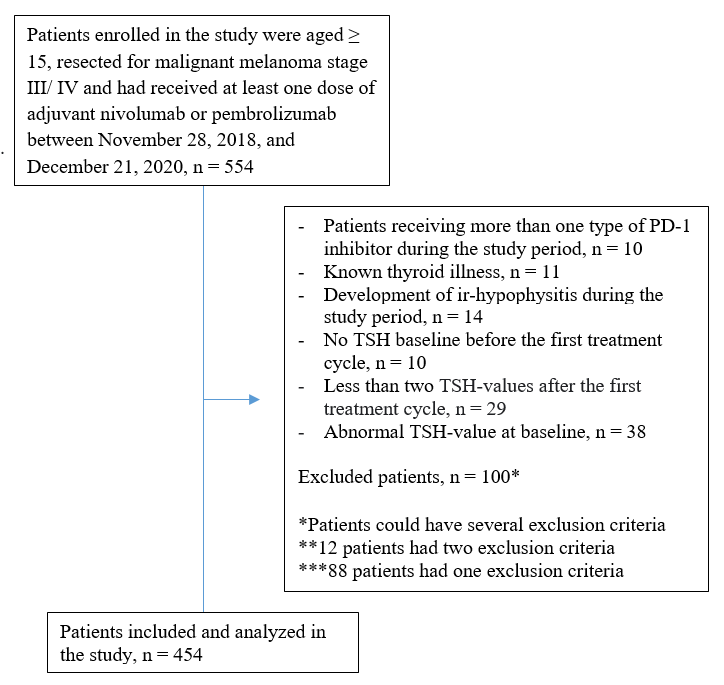


Abbreviations; ir-hypophysitis = immune-related hypophysitis, PD-1 = programmed cell death 1, TSH = thyroid stimulating hormone

Table SI1

Baseline characteristics of excluded patients due to solely missing TSH values


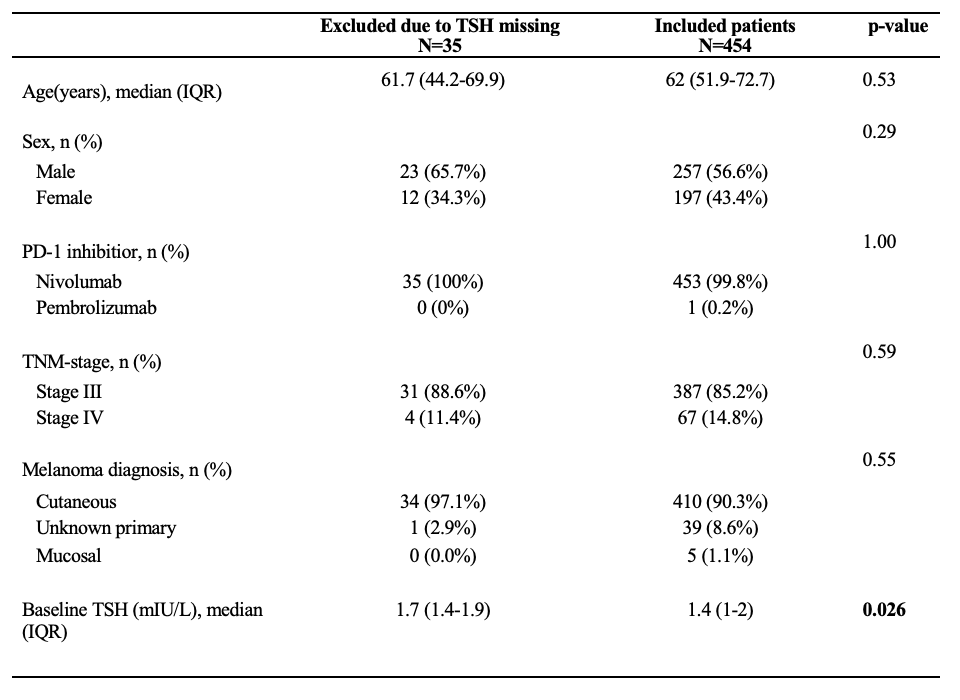


Abbreviations; PD-1 = programmed cell death 1, TNM = tumor, node, metastasis, IQR = interquartile range (25-75 % percentiles), TSH = thyroid stimulating hormone

Table SI2

Clinical characteristics of excluded patients due to solely missing TSH values


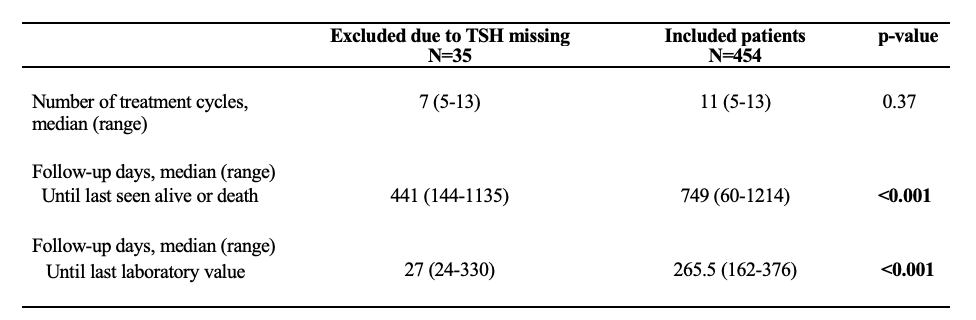


Abbreviations; TSH = thyroid stimulating hormone

Table SI3

Results of uni- and multivariate Cox Proportional Hazard Models of potential associations between age, sex and use of corticosteroid and the immune-related Thyroid Adverse Event


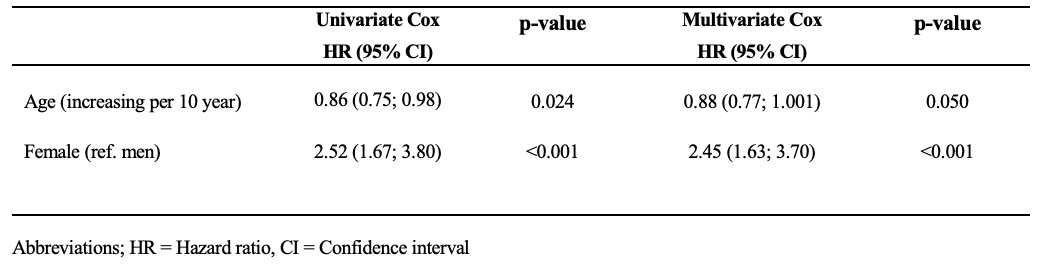


Abbreviations; HR = Hazard ratio, CI = Confidence interval
